# MR imaging

**DOI:** 10.1186/1470-7330-14-S1-O5

**Published:** 2014-10-09

**Authors:** Kartik Jhaveri

**Affiliations:** 1 University of Toronto, Abdominal Imaging,University Health Network, Mt.Sinai and Womens' College Hospital, 610 University Ave,3-957, Toronto, ON M5G 2M9, Canada

## 

Biliary tract neoplasms are commonly accounted for by cholangiocarcinoma and biliary cystadenoma/carcinoma and other less common tumors including papillary neoplasms, lymphoma and metastases. Although cholangiocarcinoma is a rare tumour (<2% of all cancer), it is the second most common primary hepatobiliary malignant tumour after hepatocellular carcinoma (HCC). This tumour actually encompasses a diverse group of tumours varying greatly in location, growth pattern and histology resulting in a gamut of imaging manifestations. It is important to be familiar with those diverse manifestations to provide accurate detection and characterization. Since only surgery can provide curative therapy, accurate resectablity assessment is critical. Defining an optimal MRI protocol which includes precontrast MR imaging along with high resolution MRCP sequences and Dynamic contrast acquisitions/MR angiography is necessary to ensure accurate results. MRI offers unique advantages via its ability to provide information noninvasively in a single test regards tumour size, extent, vascular involvement, nodes and extrahepatic spread. MRCP can superbly display bile ducts upstream to an obstruction. According to its anatomic origin cholangiocarcinoma is usually classified as intrahepatic, perihilar, or extrahepatic distal or based on growth pattern as mass forming, infiltrating or polypoidal. Staging systems have been designed for anatomical location precise for surgical planning and to establish prognosis after surgery. MRI is not without limitations. In some cases other disease process may mimic cholangiocarcinoma and these will be discussed. At times MRI may not be able to confidently detect or stage the tumor and correlative imaging with Ultrasonography, CT and PET needs to be considered. Biliary cystic tumors, such as biliary cystadenoma (BCA) and cystadenocarcinoma (BCAC) constitute <5% of all liver cysts. BCA occurs predominantly in women (90%) with mean age of 45 years while BCAC can occur equally in men and women with mean age of around 55 years. Biliary cystic tumors are commomly multilocular with thick walls and enahancing sepatations on MRI. The differential diagnosis includes hydatid cyst, liver abscess, embryonal sarcoma, primary or metastatic necrotic neoplasm, and biliary intraductal papillary mucinous neoplasm (IPMN). Lymphoma involving bile ducts is secondary to systemic lymphoma but can result in biliary obstruction that micmics klatskin tumor or inflammatory cholangitic processes. Intrabiliary metastases are most commonly due to colorectal carcinoma metastases and also occasionally secondary to lung and breast carcinoma. Intrabiliary metastases can be mistaken for cholangiocarcinoma in the absence of knowledge of a primary malignancy.
